# A *C. elegans* model for the rare human channelopathy, Timothy syndrome type 1

**DOI:** 10.17912/micropub.biology.000081

**Published:** 2018-12-18

**Authors:** Ross Lagoy, Heesun Kim, Craig C. Mello, Dirk R. Albrecht

**Affiliations:** 1 Worcester Polytechnic Institute, Department of Biomedical Engineering; 2 Worcester Polytechnic Institute, Department of Biology & Biotechnology; 3 University of Massachusetts Medical School, Program in Molecular Medicine; 4 RNA Therapeutics Institute; 5 Howard Hughes Medical Institute

**Figure 1:**
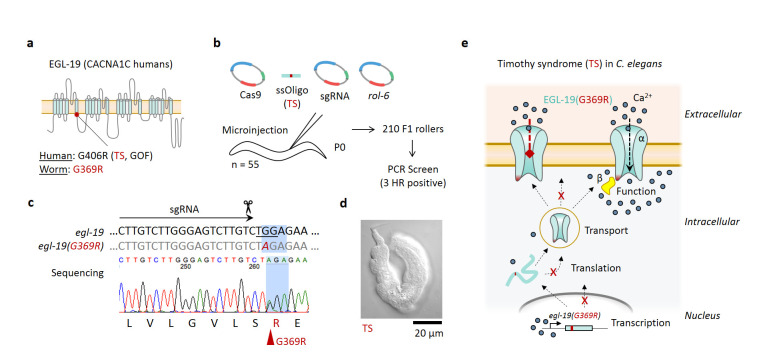
**A human Timothy syndrome mutation in the *C. elegans* voltage-gated calcium channel ortholog *egl-19* by CRISPR-Cas9 HR causes a developmental arrest phenotype.** **a.** A schematic of the human TS type 1 gain-of-function missense mutation (G406R) in the *C. elegans* ortholog EGL-19 (G369R). **b.** CRISPR-Cas9 HR mix was injected into 55 young adult *C. elegans*. Of 210 F1 roller progeny, 3 were heterozygous for the desired point mutation detected by a PCR/XbaI screen. **c.** Sequence alignment result from a HR positive heterozygous adult animal with the TS mutation. PAM sequence is underlined and small-guide RNA is beneath the arrow. Raw sequence trace result below shows the heterozygous TS A/G peak at the point mutation site (red arrow). **d.** *C. elegans* developmental arrest phenotype harboring the homozygous TS type 1 human mutation. **e.** Proposed model illustrating possible mechanisms underlying the unexpected *C. elegans* phenotype caused by the human TS type 1 mutation, which may result from worm-specific dysfunction in *egl-19* expression, trafficking, or protein function.

## Description

Timothy syndrome (TS) type 1 is a rare genetic human disease caused by a gain-of-function (GOF) missense mutation G406R in the L-type voltage-gated calcium channel (VGCC) a1 subunit gene CACNA1C, which results in severe cardiac arrhythmia and autism (Splawski *et al*. 2004). The *C. elegans* ortholog is EGL-19 (**Fig. 1a**), which is expressed in muscle and neurons (Lee *et al.* 1997). GOF mutations in e*gl-19* cause myotonic phenotypes, while reduction-of-function (ROF) mutants are flaccid, variably elongated, and egg-laying defective, and lethal mutations cause paralysis with arrested elongation at the two-fold stage (Pat) (Lee *et al.* 1997). Additionally, treatment of embryos with nemadipine-A, an antagonist of L-type VGCCs, causes a severe variable abnormal (Vab) phenotype like some ROF hatchlings (Kwok *et al.* 2006). Neural and muscle cells derived from patients with TS type 1 also yield impaired channel inactivation (Yazawa *et al.* 2011; Paşca *et al.* 2011). Therefore, we hypothesized that insertion of the human TS type 1 GOF mutation in the *C. elegans* genome (G369R) by CRISPR-Cas9 homologous recombination (HR) would cause observable changes in calcium dynamics and serve as a new animal disease model of TS to broadly investigate molecular mechanisms *in vivo*.

Instead, introduction of the TS type 1 human GOF mutation in *C. elegans* resulted in homozygous animals that closely resemble the Vab phenotype (**Fig. 1b-d**), similar to ROF mutant hatchlings and wild type embryos treated with the L-type VGCC antagonist nemadipine-A. This phenotype was observed in three independently edited lines, suggesting it resulted from the human TS mutation rather than a cis loss-of-function mutation in *egl-19* generated by the CRISPR-Cas9 editing. Further, heterozygous animals appeared wild type, which is consistent with recessive inheritance of ROF and lethal mutations in *egl-19*. Taken together, this human mutation appears to dysregulate *egl-19* function in *C. elegans* indirectly, such as through expression, trafficking, or channel kinetics at the cell membrane (**Fig. 1e**). Evaluation of gene expression, protein localization, and functional imaging or electrophysiology in this new animal model of TS type 1 are needed to distinguish among these possibilities.

This result demonstrates that CRISPR-Cas9 can be used to generate human VGCC disease mutations in *C. elegans*, although unexpected phenotypes may result from introducing human mutations in these animals. Nonetheless, this new whole organism model of TS type 1 may provide a foundation for investigating molecular mechanisms involved in this severe genetic disease, screening of additional genetic and therapeutic suppressors as potential treatments for translation, and studying other human channelopathies in *C. elegans*.

## Methods

Plasmid-based CRISPR-Cas9 homologous recombination was performed as previously described (Kim *et al*. 2014). First, a protospacer adjacent motif (PAM) was identified closest to the desired mutation site (**Fig. 1c**). Next, custom designed small-guide RNA (sgRNA) oligomers were designed and ordered (IDT) then annealed and ligated into the sgRNA plasmid backbone pRB1017 (Arribere *et al*. 2014). An 80-nucleotide single stranded oligomer (ssOligo) was custom designed and ordered (IDT) to contain the G-to-R missense mutation, which also co-acted as a silent PAM mutation and XbaI restriction site for efficient PCR screening. The final plasmid injection mix contained 50 ng/µL each of Cas9 vector, pRF4::*rol-6(su1006)* vector, sgRNA vector, and ssOligo donor. By standard microinjection technique (Mello *et al*. 1991), a total of 55 wild type animals were injected and 210 F1 animals were cloned then PCR and XbaI screened. This screen yielded three independent heterozygous HR positive lines (~2% efficiency). The G369R allele was maintained by introduction of the DnT1 genetic balancer strain (LGIV), since homozygous TS type 1 animals were unable to produce offspring. This strain (NZ1074) can be requested by contacting D.R.A. at dalbrecht@wpi.edu.
